# 

**Published:** 1918-11

**Authors:** 


### Deaths.

Dr. W. E. Hampton of Ferris, who died in Dallas, Oct. 21, had been accepted as an officer in the Medical Corps of the army and was waiting for his commission. He was prominent in medical circles of the State, was a member of Hella Temple, A. A. O. N. M. S., and belonged to the Woodmen of the World and the Odd Fellows. He was born Oct. 6, 1887, at Oxford, Miss., and was educated at the University of Mississippi and the Louisville Medical University.

Dr. Leo S. Peterson, national president of the Chi Zeta Chi medical fraternity, died of pneumonia, following influenza. Dr. Peterson was 31 years old, served for several years as captain in the New York First Field Artillery and saw service on the Mexican border as captain of the First Field ambulance.

Dr. J. R. Lewis, 31 years old, of Gainesville, died Oct. 23, at Long Island, N. Y., according to information received by relatives. According to meager and unverified reports death was due to having eaten poisoned bread some days ago. Dr. Lewis enlisted some time ago in the reserve officers medical corps and was taking special training before departing for France.

Dr. J. S. Price, one of the most prominent physicians in the State died suddenly, Sept. 13, of heart disease. He dropped dead at Hotel Dieu, where he had gone to administer to a patient.

Dr. G. J. Stapleton, of Lockney, a well known phyisician there, died of appendicitis. He was 64 years of age. He leaves a wife and several children.

The body of Munford A. Meacham, wearing the uniform of the

Medical Department of the army, was found near the tracks of the I. & G. N., near Austin, Texas, in such a position as to make investigators believe he fell from the train.

Dr. W. L. Seeger of Waxahachie died of pneumonia, Oct. 25.

Dr. C. C. Davis, Hillsboro, aged 59, died in a sanitarium at Waco, July 31, from heart disease.

Dr. Clarence LeRoy Cole, aged 41, Lieutenant-Colonel, M. C., U. S. A., San Antonio, died at his station August 9.

Dr. W. P. Breath, Galveston, aged 44, Professor of Opthalmology, University of Texas, died at St. Mary's Infirmary of apoplexy, July 26.

Dr. J. D. Stocking, Clarendon, aged 69, died of heart disease, July 18.

---

### Old Jokes That We Have Met.

A Musical Solo.—Doctor: "There must have been quite an interesting case you had in there?"

Nurse: "Yes, Mrs. Lorrey just gave birth to a 13 pound boy."

Doctor: "Was it instrumental?"

Nurse: "No vocal."—*Jour, A. M. A.*

---

Perfectly Natural.—Robust Old Gentleman (to sick woman just arrived at health resort): When I first came here I hadn't strength to utter a word; I had scarcely a hair on my head; I couldn't walk across the room, and I had to be lifted from my bed.

Sick Woman: You give me great hope. How were you cured?

Robust Old Gentleman: I was born here.

---

Babies are Old Fashioned.—"In this age of hurry and change," said Colonel Roosevelt some time ago, "I would speak a word for the old things, the conservative things. I am like the doctor—the doctor who prescribed a dose of castor oil for an ailing baby.

"The baby's fashionable young mother, who had expected the prescription to take the form of a violet ray bath or something of that kind, complained:

"'Oh, doctor, castor oil! Castor oil is such an old-fashioned remedy!'

"'Babies, madam,' said the doctor, 'are old-fashioned things.'"

---

THE COMPLAINT DEPARTMENT.—Country Lady: "I've been expecting a packet of medicine by post for a week, and haven't received it yet."

Postoffice Clerk: "Yes, madam. Kindly fill in this form, and state the nature of your complaint."

Lady: "Well, if you must know, it's indigestion."

---

THE RIGHT PLACE.—Doctor (to operetta diva who wishes to be vaccinated):—"Shall I vaccinate your arm?"

Diva: "Heavens! No, of course not. Think of me as an artist with a scar on my arm! You must vaccinate me where it won't show."

Doctor: "I think you had better take it internally."

---

Not Heard but Seen.—"Pardon me madam, but my time is not my own. You have given me all your symptoms in sufficient detail and now perhaps you will kindly let me see."

Husband: "Mathilda, he doesn't want to hear your tongue any more, he wants to look at it."

---

Too Slow.—A certain blacksmith, says Pearson's Weekly, although an expert at his trade, was quite ignorant of surgical methods. When he sprained his wrist one afternoon he hurried to a doctor's office.

The doctor examined the wrist, and then took a small bottle from a shelf, but found it empty.

"James," said he, turning to an assistant, "go upstairs and bring me down a couple of those phials."

"What's that?" exclaimed the patient suddenly, showing signs of emotion.

"I merely asked my assistant to bring me down a couple of phials from upstairs," answered the doctor.

"Files!" cried the blacksmith. "No, you don't! If that hand has got to come off, use an ax or a saw!"

---

Treatment.—"Good morning, Mrs. Gilligan; how is Patrick this morning?"

"Sure, the doctor says he's no better, sir!"

"Why don't you send him to the hospital to be treated?"

"To be treated? Faith, an' it's the delarium trimmins he has already!"

---

He Knew.—The old soldier was telling of his thrilling adventures on the field of battle to a party of young fellows, one or two of whom were skeptical as to his veracity.

"Then," he said, "the surgeons took me up and laid me in the ammunition wagon, and—"

"Look here," interrupted one of the doubtful listeners, "you don't mean the ammunition wagon; you means the ambulance wagon."

But the old man shook his head.

"No," he insisted, "I was so full of bullets that they decided I ought to go in the ammunition wagon."

